# Antidiabetic Activity of *Gomphogyne bonii* Gagnep. Extract against High-Fat Diet and Alloxan-Induced Type 2 Diabetes in Mice

**DOI:** 10.1155/2021/8648209

**Published:** 2021-11-29

**Authors:** Pham Quoc Binh, Nguyen Duy Thuan, Vu Viet Hang, Pham Thuy Phuong, Pham Quoc Su, Duong Van Phu, Phuong Thien Thuong, Dinh Thi Thu Hang, Pham Thi Van Anh

**Affiliations:** ^1^Vietnam University of Traditional Medicine, Hanoi, Vietnam; ^2^Hanoi Medical University, Hanoi, Vietnam; ^3^Vietnam-Korea Institute of Science and Technology, Hanoi, Vietnam

## Abstract

So far, diabetes mellitus has become a health threat to society all over the world. Especially, people with diabetes have always coped with complications related to this disease and unexpected side effects of synthetic drugs. Thus, there has been a current trend for researchers to find out new natural ingredients which were safer and still effective in the treatment of diabetes. *Gomphogyne bonii* Gagnep. extract (*G. bonii* extract) was an herbal-derived product of the Pharmacy Department, Vietnam University of Traditional Medicine. This study was designed to assess the antidiabetic effect of *G. bonii* extract on a high-fat diet (HFD) and alloxan-induced diabetes in mice. Mice were first fed a high-fat diet for 8 weeks and then given an intraperitoneal injection of alloxan (ALX) at the dose of 180 mg/kg b.w. After the diabetic mice model was successfully established, mice were administered orally with *G. bonii* extract at two doses of 4 mL/kg b.w/day and 12 mL/kg b.w/day for 2 weeks. The results revealed that *G. bonii* extract at both doses ameliorated the effects of ALX on the concentration of glucose, total cholesterol (TC), triglyceride (TG), and low-density lipoprotein-cholesterol (LDL-C) and microhistological images of livers. Besides, the antidiabetic effect of *G. bonii* extract at the dose of 12 mL/kg b.w/day was better than that at the dose of 4 mL/kg b.w/day. This suggested that *G. bonii* extract could be a potential agent for treating diabetes mellitus in clinical practice.

## 1. Introduction

Diabetes is a chronic, metabolic disorder as a consequence of elevated levels of blood glucose. Worldwide, the incidence of diabetes is rising rapidly and has become a community challenge, especially with low- and middle-income countries [1]. According to the IDF Diabetes Atlas (2019), the number of people with diabetes aged 20–79 years increased by 312 million (4.7% of the global population) from 2000 to 2019. It is predicted that there will be a dramatic growth in the prevalance of diabetes (about 578 million people by 2030 and 700 million by 2045) [2]. Globally, diabetes has become one of the top 10 causes of death, and the number of deaths from diabetes went up by 70% from 2000 to 2019 [3].

Due to the complications of diabetes, diabetic patients have become burdens to the families and society, leading to the health system overload. Oral antihyperglycemic drugs derived from synthetic materials were mainly used to treat diabetes, for example, sulfonylureas and biguanides. These synthetic drugs, however, caused lots of undesirable effects such as hypoglycaemia, hepatobiliary disorders, and gastrointestinal disturbances [4]. Therefore, one of the most urgent missions of research studies was the finding of novel drugs derived from herbs which not only exhibited antidiabetic effects but also limited the side effects [5].

*G. bonii* belongs to the family Cucurbitaceae, which was called “Den toong” in Vietnam. This plant has been widely distributed in northern mountainous provinces of Vietnam such as Cao Bang, Lang Son, Bac Kan, and Thai Nguyen [6]. In recent years, *G. bonii* have been widely used for the treatment of hepatitis, diabetes, cardiovascular diseases, or cancer [7]. However, so far, there have been no research studies available on the antidiabetic effect of *G. bonii* extract in the world as well as in Vietnam. Therefore, this study aimed to evaluate the hypoglycemic action of *G. bonii* extract in mice with type 2 diabetes.

## 2. Materials and Methods

### 2.1. Materials Preparation

*G. bonii* extract was provided by Pharmacy Department, Vietnam University of Traditional Medicine, Vietnam. This product achieved Standard Basis from Vietnam Institute of Materia Medica and an identification number of scientific name 18/2019 at Department of Botany, Hanoi University of Pharmacy, Vietnam.

Sample preparation was performed by multistep procedures following the standard method. Initially, all parts of herbs collected were cut into small segments of 2-3 cm long, dried, and stored in plastic bags until used. 1 kg of dried materials were extracted with 3 L of water at boiling temperature for 1 h and filtered for aqueous extracts. Notably, this step was required to repeat several times. Afterwards, the aqueous extracts were combined, evaporated in the waterbath, and adjusted to yield 1,000 mL of the extract. Thus, the *G. bonii* extract was 1 : 1 liquid extract (1 mL of liquid extract was equivalent to 1 g of materials).

### 2.2. Animal Preparation

Healthy male Swiss albino mice (weighing 21–25 g and age of 8–12 weeks) were provided by the National Institute of Hygiene and Epidemiology (NIHE), Hanoi, Vietnam. Animals were housed under the standard conditions (temperature 25°C ± 2°C and relative humidity 80% ± 10%), 12 hours dark/light time. We fed the mice with standard animal feed and allowed free access to water. After randomizing into different intervention groups as well as before implementing the experiment, mice were allowed to acclimatize for 7 days at the Laboratory of the Department of Pharmacology, Hanoi Medical University. All protocols used in this study were approved by the Scientific Board Committee of Hanoi Medical University (reference number: IRB00003121).

### 2.3. Chemical Preparation

In this study, we used alloxan 10 g (Sigma-Aldrich, Singapore) and gliclazide 30 mg (Diamicron, France). Blood glucose levels were measured by the On Call EZ II blood glucose monitoring system (ACON Biotech, USA). The biochemical analyzer (ERBA Chem., India) and commercial ERBA diagnostic kits were used for serum analysis of total cholesterol (TC), triglyceride (TG), high-density lipoprotein-cholesterol (HDL-C), and low-density lipoprotein-cholesterol (LDL-C).

### 2.4. Procedure

The study was divided into two stages following the adjusted method of Srinivasan et al. [8] and Srinivasan and Ramarao [9]:

#### 2.4.1. The First Stage

The peripheral blood test was performed on all mice in order to check fasting blood glucose concentration before starting interventions. Mice were randomly assigned to 2 groups:

  Group I (normal control) (*n* = 10): mice received normal-fat diet (NFD) (12.14% fat, 28.25% protein, and 59.81% carbohydrate)
  Group II (diabetic control) (*n* = 90): mice received high-fat diet (HFD) (42.89% fat, 18.23% protein, and 38.88% carbohydrate combined fructose syrup 55% [10]).

After 8 consecutive weeks of NFD and HFD, the changes in blood glucose levels of all mice were recorded and compared among groups. Mice at group 2 were injected i.p with alloxan (ALX) at the dose of 180 mg/kg, while the NaCl 0.9% injection was given for group I. After 72 hours of the injection, all mice were examined for blood glucose levels (*t*_0_). Mice with fasting blood glucose levels exceeding 8.0 mmol/L were considered diabetic.

#### 2.4.2. The Second Stage

The study was carried out in a continuous 14-day period. All diabetic mice determined at the first stage were randomly separated into groups (from group 2 to group 5). As a result, at this stage, there were 5 groups with 10 mice per group, and the intervention was performed as followed:

  Group 1 (control): NFD + distilled water
  Group 2 (model): HFD + an injection of ALX 180 mg/kg + distilled water
  Group 3 (gliclazide): HFD + an injection of ALX 180 mg/kg + gliclazide at the dose of 80 mg/kg/day
  Group 4 (low-dose *G. bonii* extract): HFD + an injection of ALX 180 mg/kg + *G. bonii* extract at the dose of 4 mL/kg/day
  Group 5 (high-dose *G. bonii* extract): HFD + an injection of ALX 180 mg/kg + *G. bonii* extract at the dose of 12 mL/kg/day

After 1 week (*t*_1_) and 2 weeks (*t*_2_) of treatment, the fasting blood glucose levels in mice were tested. At the end of the experiment, all animals were checked blood lipid indexes (total cholesterol (TC), triglyceride (TG), HDL-C, and LDL-C) and subjected to a full gross necropsy. In 30% of mice of each group, liver and pancreas were removed for histopathology examinations.

### 2.5. Statistical Analysis

Data were analyzed using IBM SPSS Statistics for Windows, version 20.0 (IBM Corp., Armonk, N.Y., USA). The *t*-test and ANOVA tests were performed to examine the differences in blood glucose levels and serum lipid profiles in different groups. Data were shown as $\bar{X}$ ± SD. A *p* value <0.05 was considered to be statistically significant.

## 3. Results

### 3.1. Effect of *G. bonii* Extract on the Weight of Mice

**Figure 1:**
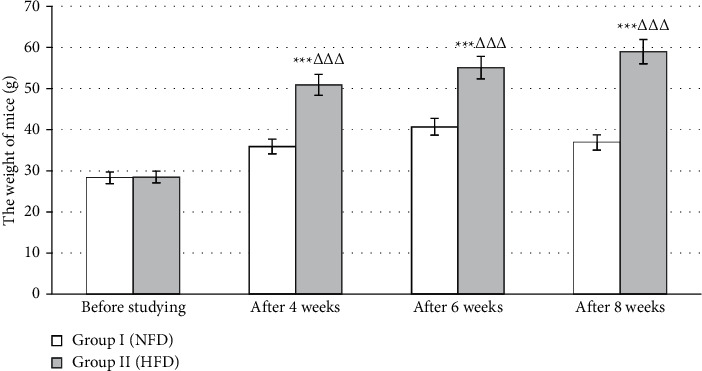
The change in the weight of mice. NFD, normal-fat diet; HFD, high-fat diet. ^∗∗∗^*p* < 0.001 compared with the weight before studying. ^ΔΔΔ^*p* < 0.001 compared with the weight of group fed with NFD.

As can be seen from Figure 1, after 4 weeks, 6 weeks, and 8 weeks, the weight of mice at the group fed with HFD increased dramatically as compared with that before studying (*p* < 0.001). Moreover, at all time points, there was a substantial rise in the weight of mice at the group fed with HFD as compared with the group fed with NFD (*p* < 0.001).

### 3.2. Effect of *G. bonii* Extract on Blood Glucose Levels

As given in Table 1, after 72 hours of ALX injection, at group II, there was an upward trend in the blood glucose levels as compared with time points “Before studying” and “After 8 weeks” (*p* < 0.001). Besides, after 8 weeks of HFD and after 72 hours of ALX injection, the blood glucose levels at group II posed a significant development as compared with group I (*p* < 0.01 and *p* < 0.001, respectively).

After 1 week and 2 weeks of treatment, *G. bonii* extract at both doses of 4 mL/kg/day and 12 mL/kg/day administrated orally reduced remarkedly blood glucose levels as compared with the model group (*p* < 0.05 and *p* < 0.01, respectively). No significant difference was observed in blood glucose levels between groups treated *G. bonii* extract and gliclazide (*p* > 0.05) (Table 2).

### 3.3. Effect of *G. bonii* Extract on Blood Lipid Concentrations

Table 3 presents lipid disorders of group 2 (model) through the high concentration of TC, TG, and LDL-C. At the group treated with *G. bonii* extract at the dose of 4 mL/kg/day, there was a downward trend in TC and LDL-C concentrations as compared with group 2, but no significant difference was observed (*p* > 0.05). *G. bonii* extract at the dose of 12 mL/kg/day decreased considerably TC, TG, and LDL-C concentrations as compared with group 2 (*p* < 0.05). No significant difference, however, was observed between the groups treated with Gb extract and group 2 in terms of HDL-C concentration (*p* > 0.05).

### 3.4. Effect of *G. bonii* Extract on Microhistopathological Study of the Liver and Pancreas

**Figure 2:**
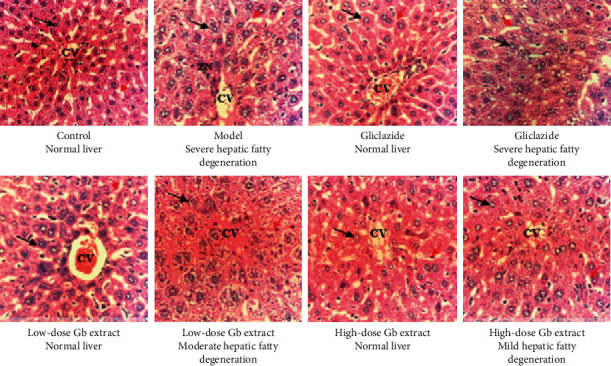
Microhistopathological images of the liver (HE × 400). CV, central venule; ZN, zonal necrosis; Gb, *G. bonii*. The black arrow indicates hepatocytes, and the red arrow indicates sinusoids.

**Figure 3:**
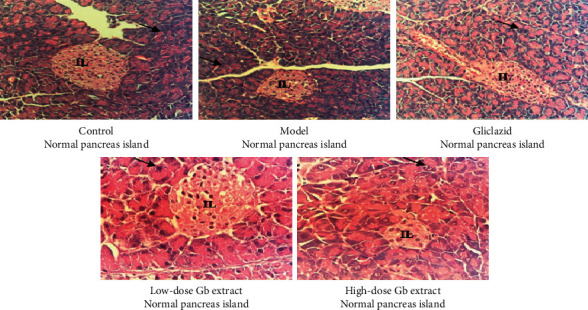
Microhistopathological images of the pancreas (HE × 400). IL, the islet of Langerhans; Gb, *G. bonii*. The black arrow indicates acinar cells.

As shown in Figures 2 and 3, severe hepatic degeneration was found with necrosis zones at group 2 (model). Gliclazide and *G. bonii* extract at both doses of 4 mL/kg/day and 12 mL/kg/day improved markedly histopathological examination of livers in comparison to the model group. Several images of livers at groups treated with gliclazide and *G. bonii* were added to illustrate the recovery of the hepatic degeneration as compared with the model group (group 2). In particular, some mice had the normal structure of livers. No significant difference was observed in histopathological images of the pancreas between groups treated with *G. bonii* extract and group 2.

## 4. Discussion

The main pathogenesis of type 2 diabetes has been associated with peripheral insulin resistance and deficient insulin production due to the dysregulation of insulin secretion. It was concluded that obesity was one of the main risk factors in insulin resistance [11]. Besides, in experimental studies, alloxan was one of the most common agents to induce the hyperglycemia mice model [10]. Through destroyed *β*-cells of the pancreas, insulin release was inhibited by alloxan, leading to hyperglycemia [12, 13]. Thus, we used alloxan-combined high-fat diet as agents to induce the diabetes model.

As a result, after 4 weeks, 6 weeks, and 8 weeks of the first stage, the weight of mice increased by 78.8%, 93.4%, and 107.1% at group II fed with HFD and only 34.9%, 43.8%, and 30.4% at group I fed with NFD as compared with the time point “Before studying.” In terms of the blood glucose levels, after 8 weeks of having HFD, there was a considerable rise as compared with the group fed with normal-fat diet (NFD). After 72 hours of ALX injection, at the group fed with HFD, the blood glucose levels increased substantially and was approximately 1.5 times higher than that of mice before studying. Thus, the HFD-combined ALX injection was proved to have an effect on hyperglycemia and bring into success in the model of type 2-like diabetes.

After 1 week and 2 weeks of treatment, gliclazide at the dose of 80 mg/kg/day and *G. bonii* extract at both doses of 4 mL/kg/day and 12 mL/kg/day reduced statistically blood glucose levels in type 2-like diabetic mice as compared with the model group. Moreover, *G. bonii* extract posed a positive effect on lipid disorder conditions. *G. bonii* extract at the dose of 12 mL/kg/day reduced significantly TC, TG, and LDL-C concentration as compared with the model group. Besides, *G. bonii* extract at the dose of 4 mL/kg/day only tended to decrease TC and LDL-C concentration as compared with the model group.

The result of histopathological examination showed that there was a significant improvement in the structure of the liver after the 2-week period at the groups treated with *G. bonii* extract. In terms of histopathological images of the pancreas, no clear effect was observed in the groups treated with *G. bonii* extract as compared with the model group.

These results indicated that *G. bonii* extract had the antidiabetic effect in high-fat diet and alloxan-induced diabetic mice. This was due to the effect of the main component (*G. bonii*) in this product. In recent years, *G. bonii* was used empirically to treat diabetes in clinical treatment and posed beneficial effects for diabetic patients [7]. *G. bonii* was a newly discovered plant from Cucurbitaceae family which is a family of herbaceous plants or the gourd family of flowering vines belonging to the order Cucurbitales. Various plants from Cucurbitaceae family posed an antihyperglycaemic effect in alloxan-induced diabetic mice [14].

## 5. Conclusion

In light of these findings, oral administration of *G. bonii* extract at both doses of 4 mL/kg/day and 12 mL/kg/day for 2 consecutive weeks exhibited significant antidiabetic activity through reducing blood glucose levels and plasma lipid profiles (TC, TG, and LDL-C concentration) and improving hepatocytes injury in the high-fat diet and alloxan-induced diabetes model. Moreover, the high dose (12 mL/kg/day) had more potent antidiabetic properties than the low dose (4 mL/kg/day). What is more, in view of the current lack of knowledge regarding *G. bonii*, further research studies about the mechanism of the antidiabetic effect of *G. bonii* are really needed and highly recommended.

## Figures and Tables

**Table 1:** The change in blood glucose levels of mice.

Time	Blood glucose levels (mmol/l) ($\bar{X}$ ± SD) (*n* = 10)		*p* (compared with group I)
	Group I: NFD	Group II: HFD	
Before studying	4.80 ± 0.88	5.25 ± 1.42	>0.05
After 8 weeks	4.95 ± 0.85	7.13 ± 1.96	<0.01
72 hours after ALX injection	5.35 ± 0.65	10.60 ± 2.76^*∗∗∗*+++^	<0.001

NFD, normal-fat diet; HFD, high-fat diet. ^∗∗∗^*p* < 0.001 compared with the time point “Before studying.” ^+++^*p* < 0.001 compared with the time point “After 8 weeks.”

**Table 2:** Effect of *G. bonii* extract on blood glucose levels after 2 weeks of treatment.

Group	Blood glucose levels (mmol/l) ($\bar{X}$ ± SD) (*n* = 10)		
	*t*_0_ (before treatment)	*t*_1_ (after 1 week of treatment)	*t*_2_ (after 2 weeks of treatment)
Group 1 (control)	5.35 ± 0.65	5.96 ± 0.84	5.70 ± 0.76
Group 2 (model)	11.10 ± 2.21^∗∗∗^	11.00 ± 2.98^∗∗∗^	9.58 ± 2.24^∗∗∗^
Group 3 (gliclazide 80 mg/kg/day)	10.67 ± 1.94^∗∗∗^	8.40 ± 1.17^Δ^	7.59 ± 1.34^Δ^
Group 4 (low-dose *G. bonii* extract)	10.10 ± 2.45^∗∗∗^	8.44 ± 1.98^Δ^	7.76 ± 1.14^Δ^
Group 5 (high-dose *G. bonii* extract)	10.12 ± 2.37^∗∗∗^	7.49 ± 1.32^ΔΔ^	7.05 ± 1.29^ΔΔ^

^∗∗∗^ *p* < 0.001 compared with the control group. ^ΔΔΔ^*p* < 0.05 and *p* < 0.01 compared with the model group.

**Table 3:** Effect of *G. bonii* extract on blood lipid concentration after 2 weeks of treatment.

Group	Blood lipid concentration (mmol/L)			
	TC	TG	HDL-C	LDL-C
Group 1 (control)	2.44 ± 0.30	0.86 ± 0.18	0.87 ± 0.11	1.18 ± 0.21
Group 2 (model)	2.88 ± 0.42^*∗*^	1.09 ± 0.13^∗∗^	0.86 ± 0.12	1.52 ± 0.41^*∗*^
Group 3 (gliclazide 80 mg/kg/day)	2.72 ± 0.15	0.95 ± 0.10^Δ^	0.91 ± 0.17	1.38 ± 0.24
Group 4 (low-dose *G. bonii* extract)	2.72 ± 0.28	1.19 ± 0.22^Δ^	0.83 ± 0.13	1.35 ± 0.25
Group 5 (high-dose *G. bonii* extract)	2.36 ± 0.37^ΔΔ^	0.94 ± 0.18^Δ^	0.79 ± 0.19	1.14 ± 0.36^Δ^

^∗∗∗^ *p* < 0.05 and *p* < 0.01 compared with the control group. ^ΔΔΔ^*p* < 0.05 and *p* < 0.01 compared with the model group. TC, total cholesterol; TG, triglyceride; HDL-C, high-density lipoprotein-cholesterol; LDL-C, low-density lipoprotein-cholesterol.

## Data Availability

The data used to support the findings of this study are available from the corresponding author upon request.
